# Boston Letter

**Published:** 1879-05

**Authors:** 


### Domestic Correspondence.

### ARTICLE XIII.

### BOSTON LETTER.

*Messrs. Editors* :—Probably there is no city in the country in which surgeons and physicians are so unanimous in the use of ether as an anæsthetic, and so determined to have nothing to do with chloroform, as in Boston. It may be said that this condition of things is perfectly natural, Boston being the city which introduced ether to the profession. But I think this enmity to chloroform springs rather from the habitual conservatism of our physicians. They see that ether is a safe anæsthetic; they see that the administration of chloroform is attended with danger and therefore let it alone.

There are a very few exceptions in which our physicians give chloroform in confinement cases, but I cannot remember that I ever saw it used in the hospitals of this city. Boston surgeons do not relish waiting for the insensibility of an etherized patient any more than do the surgeons of other cities, but they prefer to wait rather than run risks. There are surgeons who do not seem to consider that life is of more value than time. Since the day when Dr. John Collins Warren performed the first operation upon an etherized patient—now thirty-three years ago—Boston surgeons have found ether good enough for their purposes. At the Massachusetts General Hospital the administration of ether has been reduced to a perfect system. The patients are etherized long enough in advance of the operation to preclude all waiting on the part of the surgeons. After the operation, unless their recovery from the ether intoxication be complete, they are not taken back into the wards, but are conveyed to the "recovering rooms," which adjoin the amphitheater, and there remain until the fight and delirium have disappeared. This is an exceedingly wise arrangement, for the patients in the wards are thereby protected from the effects of fear or nervousness invariably caused by men who are half wild from the fumes of ether. Moreover, there is in this amphitheater an operating chair, a most ingenious affair, devised by Dr. H. J. Bigelow in 1854, by means of which an etherized patient is not only perfectly secured and incapable of movement during an operation, but if syncope come on, the patient's head can instantly be dropped below the level of the body merely by withdrawing a bolt. It was the necessity of having some way of accomplishing this sudden change of position which led Dr. Bigelow to invent the chair. It likewise enables the surgeon to place his patient in any desired position. By its means, too, much less assistance is required during operations, for the patient being powerless no men are needed to hold the limbs during the excitement of the etherization. A similar chair is now in use at the City Hospital; a third is the property of Dr. Carleton, of Connecticut; a fourth was procured for the New York Hospital, by Dr. Sands; a fifth copy was made for the Empress of Germany, who heard of it from Dom Pedro, who saw the chair while he was in Boston—the Empress sent for photographs of it in order that it might be used in hospitals in Germany in which she is greatly interested. It should be in every operating amphitheater in America, and can be ordered from Codman & Shurtleff, of this city. In connection with the use of ether at the Massachusetts General Hospital, I should mention that in one of the instrument cases in the amphitheater is the first sponge ever used for etherization, a souvenir of the original painless operation, by Dr. J. C. Warren in 1846. Naturally it is an interesting and precious relic. A few years ago Dr. B. Joy Jeffries, of this city, went to England with the purpose of demonstrating the superior safety of ether and of converting English surgeons to its use. In many cases he succeeded, but chloroform is still the common anæsthetic in that country, and we are constantly hearing of deaths which have been the result of its use. The fact that chloroform was introduced by a British physician—Simpson, of Edinburg, in 1847—may have some influence in shutting out ether. But the protests against chloroform which appear from time to time in British journals indicate that Dr. Jefries' leaven is working. Fortunately, he took several cans of good American ether with him, otherwise, unless English ether is now of better quality than formerly, I fear his success would have been less striking. In 1872 I attended a confinement case in London, during which I administered twenty ounces of the best English ether I could obtain, yet could not for one moment make my patient fully insensible. Must I add that, notwithstanding Dr. Jeffries clearly stated the purpose for which he had brought it, the English Custom House authorities *obliged him to pay duty upon his American ether!* This was next to putting a premium upon narrow-mindedness. Many physicians in Great Britain and elsewhere will doubtless be influenced by Balfour's sturdy recommendation of chloroform in his recent work on "Diseases of the Heart." Balfour considers it the remedy *par excellence* in angina pectoris, and I know of one prominent Boston physician who thinks likewise. Balfour, however, literally staggers one by saying not only that "it is *perfectly safe*" but, if so arranged as to be inhaled only in small doses, that "*it is not even necessary for a medical* man to administer it." He thinks that before the patient can inhale a large quantity the bottle will fall from his hand. From a man so generally trustworthy as Balfour such advice is amazing. I find that if a physician has had the pleasure of bringing back to life one case of chloroform syncope, he is glad thereafter to confine himself to ether. I once had that experience, and it is a frightful one, which will never be repeated at my hands. In this country I suppose Prof. Gross, of Philadelphia, is our most vigorous defender of chloroform; although he has had some startling experiences; he will use no other anæsthetic. But in spite of his strong influence the majority of Philadelphia surgeons, one might say nearly every one of them, use ether and fear chloroform. Just now, going the rounds of the medical press, the conclusions of Vergely, of Bordeaux, are newly calling attention to chloroform. This gentleman concludes that heart-disease does not contra-indicate its use; that it is a sedative in this class of diseases; that it should be used with discretion. His last clause is the only sensible one of the three, though the second, unfortunately, is but too true in a sense more sad than Vergely means to convey. He further thinks this agent is given too timidly and unsystematically in angina pectoris and affections characterized by dyspnœa and palpitation. When one considers the history of chloroform and the number of deaths it has caused and is causing, a feeling of strong indignation is the result of reading such advice. We can only hope it may not be followed. Need I apologize for this dissertation, the length of which has been quite involuntary? I cannot easily avoid being an assailant when I see chloroform extolled by such men as Balfour, Berkeley Hill and others as perfectly safe, "*safer*," says Hill, "*than ether.*" It seems to me, too, that those who realize the dangers of chloroform narcosis are not sufficiently outspoken.

You are aware, perhaps, that a system of "intersecting sewers" is now being constructed in Boston. The cost will amount to millions but the necessity was great, for imperfect drainage was committing ravages in the best sections of the city. The city engineer says that drains and sewers are constantly being uncovered whose age and primitive character are surprising. Many of them were built for single houses and have been in existence since the early portion of the century. Their condition, in frequent instances, is shockingly bad. It will interest you to know what the new system of sewerage is and what it will probably accomplish, but this matter for the present may be kept in reserve. My reference to it now is merely preliminary to mention of the somewhat unexpected revelations of the books of our Board of Health. In view of our imperfect drainage, of general complaints, in certain winds, of the presence of sewer gas in house and atmosphere, in view, too, of the accepted theory that sewer poison is a common cause of typhoid fever, we naturally might look for a large death rate from that disease. I was, therefore, surprised to observe that 1370 cases of diphtheria were reported during the year 1878 and 450 deaths, while only 120 deaths were caused by typhoid fever. The question at once arises, may we not find that sewer emanations expose us to the greater danger of diphtheria more certainly than to typhoid fever? In some instances in this city, where the patients have been treated by prominent physicians, it has been discovered that the cause of fatal diphtheria proceeded from imperfect soil pipes. Many isolated cases have left it impossible to deny that foul gases have been the cause of this dangerous disease. If it be true that the cases which occur in Boston are the result of foul air, we can only hope there may be more typhoid and less diphtheria until the day comes when good sewerage will relieve us from both. But I have also remarked that in Boston the death rate of diphtheria for 1878 was very low, viz., 30¼ per cent. It may, therefore, be hoped, since homœopaths call every cheesy follicle diphtheria, that of the 1,370 cases reported many, let us say hundreds, were not diphtheria at all, but only follicular sore throat or tonsillitis; 1,370 cases and only 450 deaths is not the usual proportion in true diphtheria. Of scarlet fever there have been comparatively few cases, and only 68 deaths by that disease were reported during the year 1878. Our Board of Health has been unceasing in its endeavors to enlighten the people in regard to the various forms of scarlet fever. In its circular which is habitually sent to families in which this disease is reported, there is the wise warning: "Scarlet fever, scarlatina, canker-rash and rash-fever are names of one and the same dangerous disease." Moreover, the circular strongly impresses the fact that scarlet fever resembles small-pox in its power to spread rapidly from person to person. The care and arrangement of the sick-room are clearly set forth, and very sensible directions are given in regard to the period of convalescence and disposition of bedding and clothing which have been worn during the illness. The proper manner of purifying the room is likewise explained, and finally the advice is given that in case of death the funeral services should be strictly private and that the body should not be exposed to view.

A few weeks ago one of these useful and sensible circulars was returned to our Board of Health by a homœopath who had found it at the house of a patient ill from scarlet fever. The physician had enclosed the sections relating to the convalescent period, care of clothing and room, and the conduct of funerals, in brackets, with the marginal note, "Nonsense"! He had endorsed the circular as follows: "Sent to a patient of the undersigned. I will look out for the cleansing. C. F. N." This astute gentleman, I regret to say, is a graduate of the Harvard school; but since he made use of that school merely to give himself some preparation for practice, meanwhile fully intending to be a homœopath in name, it can easily be seen that even as a student he made the discovery that he knew more than either his teachers or his books, and this discovery, evidently, is the guide of his life. It is such men as he who render the control of scarlet fever difficult, and who aid in spreading the contagion.

Another extremely wise step has been taken by our Board of Health. When it becomes known at headquarters that a person is ill of scarlet fever, the Board at once excludes from school every child in the family in which the disease has occurred. The enforced absence (save in exceptional cases) continues four weeks from the beginning of the attack, and when the scholar is re-admitted to the schoolroom, it must be on the certificate of a physician that the required time has passed. Such a course has been adopted because the Board of Health realizes that perfect quarantine is the only means by which scarlet fever can be stamped out. Of course the quarantine enforced upon families is not perfect, and for obvious reasons. But in his earnestness and thoroughness, Dr. Durgin, the genial chairman of the Board, if permitted, would have every house in which scarlet fever existed guarded by a policeman whose duty it should be to see that no one but the physician entered or left it until quarantine was removed. Supplies of food would be left at the door. To the half-hearted neutralists who, upon the whole, think scarlet fever is not particularly contagious, such stringent regulations would appear excessive, but it is a sad truth that lives are lost every year by the criminal negligence of those who come in contact with scarlet fever. This applies to physicians as well as laymen. The fixed rule of every physician should be to visit his scarlet fever patients last of all. Upon reaching his house he should take a bath and change his outer garments, hanging in the open air for several hours those he has just put off. He should likewise quarantine himself in his office, and take his meals and sleep there until he has done with scarlet fever. When a physician falls into an error of judgment, his mistakes should always be made upon the safe side, especially when questions arise concerning the proper care of contagious affections and the protection of those who are exposed to them. * *

BOSTON, April, 1879.

